# Baseline differences in characteristics and risk behaviors among people who inject drugs by syringe exchange program modality: an analysis of the Miami IDEA syringe exchange

**DOI:** 10.1186/s12954-019-0280-z

**Published:** 2019-01-23

**Authors:** Siddharth Iyengar, Adam Kravietz, Tyler S. Bartholomew, David Forrest, Hansel E. Tookes

**Affiliations:** 1 Department of Public Health Sciences, University of Miami Miller School of Medicine, Miami, FL USA; 2 Department of Anthropology, University of Miami College of Arts and Sciences, Coral Gables, FL USA; 3 Department of Medicine, Division of Infectious Diseases, University of Miami Miller School of Medicine, Miami, FL USA

## Abstract

**Background:**

In March of 2016, Florida passed the Infection Disease Elimination Act (IDEA), legalizing the formation of the first syringe exchange program in Florida, which opened in December of 2016 at a fixed site in Overtown, Miami. Since that time, the exchange expanded in April of 2017 to include a mobile van unit that provides the same services at different locations throughout Miami-Dade County.

**Methods:**

Trained interviewers conducted face-to-face interviews from all first-time participants at the IDEA Exchange, both at the fixed site and the mobile van unit.

**Results:**

Among 718 first-time enrollees, 74.8% were male, 52.1% were non-Hispanic White, 85.9% completed high school, 59.8% were unemployed, 42.1% were homeless, 54.2% reported an annual income of less than $15,000, and the mean age was 38 years. Participants at the fixed site and mobile van unit reported differences in socioeconomic status, injection drug-related behaviors, and pre-existing hepatitis C virus (HCV) infection status.

**Conclusions:**

Taken together, these results suggest that the mobile unit is capturing a subset of PWID in Miami that the fixed site is not, and vice-versa. As the opioid crisis extends into all demographics, such multimodal efforts to target various populations of PWID should be kept in mind, especially when unveiling future syringe exchanges in Florida and other late-adopting states.

## Background

Multiple studies of syringe exchange programs (SEPs) in the USA and abroad have found SEPs to be associated with decreases in high-risk injection drug use behaviors without facilitating drug use [1–5]. SEPs have been found to be cost-effective ways to reduce the spread of communicable disease due to injection drug use, especially HIV [5, 6]. SEPs have been utilized in major cities throughout the world since the 1980s after the introduction of the highly successful SEP in Amsterdam in 1984 [7]. In North America, success was seen with SEPs in the 1990s in cities such as New York City, Vancouver, and Baltimore and was highlighted by the US Surgeon General at that time [8]. Much attention has again been refocused on SEPs in the USA due to the current opioid crisis, which has been declared a national public health emergency.

In 2015, the Centers for Disease Control and Prevention (CDC) released its annual HIV Surveillance Report, which showed that Florida had the highest number of new HIV and AIDS diagnoses in 2014 of any state [9]. Data from the Florida Department of Health showed that 23% of these HIV diagnoses and 19% of these AIDS diagnoses occurred in Miami-Dade County [10]. Additionally, CDC data showed that Miami had the highest incidence of HIV in the USA in 2014 among all US cities and every subsequent year to date [9, 11, 12]. Studies also showed that the burden of HIV in Miami was heavily driven by injection drug use [13–15]. In light of this, the state of Florida passed the Infection Disease Elimination Act (IDEA) in March of 2016, which legalized the formation of a 5-year pilot syringe exchange program to be established in Miami-Dade County under the operation of the University of Miami. The program, as stipulated by law, is privately funded and operates using a strict 1:1 exchange model. This syringe exchange is the first of its kind in the state of Florida and became operational in December of 2016 at a fixed location Overtown, a predominantly underserved African-American neighborhood of Miami known for high rates of illicit drug activity and overdose deaths.

Responding to an increased demand for its services, the IDEA Exchange expanded via a mobile van unit, which became operational in April of 2017. While the mobile van unit has been stationed in multiple locations throughout Miami-Dade County, it primarily provides its services in Overtown, the same community as the fixed site. Previous data on mobile unit syringe exchange has shown that they can offer an additional modality with which to target hard-to-reach people who inject drugs (PWID) [16, 17]. Specifically, while the World Health Organization, Joint United Nations Programme on HIV/AIDS, and United Nations Office on Drugs and Crime have recommended the use of mobile SEPs, a recent critical review of mobile SEPs showed that the evidence regarding their efficacy is lacking [18]. The purpose of this study was to examine differences in sociodemographic factors, injection drug use behaviors, sexual risk behaviors, and HIV/HCV status between those surveyed at the mobile van unit and the fixed-site syringe exchange in order to better inform implementation of SEPs in late adopting states such as Florida.

## Methods

This study analyzed data from a 42-item enrollment survey developed by the study’s authors in conjunction with experts from other SEPs in the USA as well as peer educators and outreach workers who have experience working with PWID. These surveys assessed participant demographics, injection drug use behaviors, sexual behaviors, and HIV/HCV indicators. This analysis was submitted to the University of Miami Institutional Review Board (Study #20160932) and determined not to be human subjects research.

### Study population

Participants were a convenience sample of PWID who visited either the fixed site of the IDEA Exchange in Overtown, Miami, or the mobile van unit to enroll in the program. Participant eligibility criteria included enrollment in the IDEA Exchange program, age of 18 years or older, and ability to complete the interviewer-administered survey in English or Spanish. Enrollment surveys were administered prior to provision of harm reduction services. This study includes all 718 participants enrolled through April 2018 at the fixed site (591 participants) since its inception in December 2016 as well as the mobile van unit (127 participants) which became operational in April 2017. Sites of recruitment for the mobile van unit include weekly visits to the following Miami-Dade County cities/communities: Overtown, Liberty City, Florida City, Opa-Locka, Miami Beach, and Miami Gardens. No monetary incentives were provided for enrollment in the program.

### Study design

The study’s authors developed survey questions with input from experts at other harm reduction services as well as peer educators and outreach workers (i.e., individuals with expertise in SEP service provision as well as working with PWID) to design the 42-item survey and to train interviewers. Interviewers consisted of students and IDEA Exchange staff who underwent training to ensure standardized administration of the survey. Surveys were orally administered in a face-to-face interview and took approximately 15 min to complete. The interviews were conducted in private rooms either in the mobile van or the fixed site. No personal identifying information was collected from participants. Participants were assigned a four-digit unique identifier and given an identification card containing only this number that is used to keep records. These surveys assessed participant demographics, injection drug use behaviors, and HIV/HCV indicators, as required by state statute. Survey data was collected on electronic tablets using REDCap® software. Upon completion of the survey, participants were able to participate in 1:1 exchange of syringes, receive sterile injection equipment, obtain an on-site HCV/HIV test, and were counseled on HCV/HIV prevention and harm reduction practices.

### Measures

The outcomes for this study included any differences in baseline characteristics between PWID at the fixed site or the mobile van unit. Demographic data assessed participants’ age, gender, race/ethnicity, marital status, sexual orientation, highest level of educational attainment, employment status, annual household income, housing status, insurance status, ZIP code of residence, and method in which they heard about the exchange. Based on responses from participants’ ZIP code of residence, their county of residence was inferred and recoded into “Miami-Dade,” “Broward,” or “Other.” For method by which participants found out about the exchange, participants were asked “Where did you hear about the IDEA Exchange?” and were presented the options of “Word of mouth,”, “Online,” “Fliers/newspaper,” or “Referral from healthcare provider,” from which they were allowed to make multiple selections.

For drug use behaviors, participants were asked at what age they first started using drugs, what drugs they injected in the past month, if they used prescription opioids prior to ever injecting drugs, how many times per day they injected drugs on average during the past month, and the location they most often injected during the past month. Injection drug use in the past month was assessed for the following substances: heroin, cocaine, speedballs (heroin and cocaine), methamphetamine, fentanyl/carfentanil, crack, painkillers/opioids, and any other drugs not listed. Additionally, participants were asked how often during the last month they used alcohol swabs or reused or shared needle/injection equipment when injecting. Responses for these questions utilized a 4-point Likert scale, with choices including “None of the time,” “Some of the time,” “Most of the time,” or “All of the time.” The response “None of the time” corresponded to participants’ never practicing this behavior during the past month, “Some of the time” as less than 50% of the time when practicing this behavior, “Most of the time” as greater than 50% of the time when practicing this behavior, and “All of the time” as each time when practicing this behavior. Additionally, to assess overdose behavior, participants were asked if they had ever overdosed due to injection drug use. Those who had reported an overdose were asked the number of times they had overdosed, and an average number of times overdosed for these participants was calculated from this data.

For self-reported HIV/HCV status, participants were asked for their prior HIV/HCV status—negative, positive, or unknown. All participants were offered a fingerstick test for HIV using the OraQuick Advance® Rapid HIV-1/2 Antibody Test and a fingerstick test for HCV using the OraQuick® HCV Rapid Antibody Test. Results were recorded as positive or negative. Those who identified as HIV-positive at baseline were asked if they were currently receiving HIV treatment.

For each question, participants were offered the option to refuse to answer, for which these responses were not included in the analysis.

### Statistical procedures

Chi-square tests were used to evaluate the differences in sample distribution between the fixed site and mobile van unit. Fisher’s exact test was used for variables containing responses with small sample sizes. Post hoc pairwise comparisons were performed for subgroup analyses using the Bonferroni correction (*p* < 0.005). Statistical significance was defined by *p* values < 0.05. Statistical analyses were performed using Stata® v.12 (Stata Corp., College Station, TX).

## Results

Seven hundred eighteen participants were enrolled during the study period, of which 591 (82.3%) were enrolled at the fixed site and 127 (17.7%) at the mobile van unit. Among all enrollees, 74.8% were male, 52.1% were non-Hispanic White, 85.9% completed high school, 59.8% were unemployed, 42.1% were homeless, 54.2% reported an annual household income of less than $15,000, and the mean age was 38 years. Compared to participants enrolled at the fixed site, a greater percentage of PWID at the mobile van unit were female (35.4% vs 22.8%, *p* = .002), non-Hispanic Black/African-American (18.1% vs 4.9%, *p* < .001), currently homeless (61.7% vs 38.7%, *p* < .001), and had been unemployed for greater than 1 year (43.5% vs 31.5%, *p* = .031) (Table 1). Fifty-one percent of PWID enrolled at the mobile van unit reported an annual household income of less than $5000, compared to 29.1% of PWID enrolled at the fixed site (*p* < .001). Word of mouth was the most reported method of hearing about the exchange at both sites (80.6% mobile van unit vs 67.4% fixed site). However, many more fixed site enrolled participants reported hearing about the exchange online (8.2% mobile van unit vs 26.9% fixed site, *p* < .001). Fewer mobile van unit enrolled participants were from Broward County (10.3% mobile van unit vs 19.6% fixed site, *p* = .031). The two groups did not differ significantly by age, educational attainment, marital status, or sexual orientation.

**Table 1:** Sociodemographic characteristics among participants at the Miami IDEA syringe exchange

Characteristics	Sample total *n* (%)	Fixed site *n* (%)	Mobile van unit *n* (%)	*p* value
Total	718 (100.0)	591 (82.3)	127 (17.7)	
Age				
Mean ± standard deviation	38.44 ± 11.04	38.13 ± 10.80	39.92 ± 12.06	0.124
18–24	37 (5.2)	30 (5.1)	7 (5.5)	0.611
25–29	132 (18.5)	111 (18.9)	21 (16.5)	
30–39	266 (37.2)	224 (38.1)	42 (33.1)	
40–49	162 (22.7)	131 (22.3)	31 (24.4)	
50–59	72 (10.1)	58 (9.9)	14 (11.0)	
≥60	46 (6.4)	34 (5.8)	12 (9.4)	
Gender				
Male	537 (74.8)	456 (77.2)	81 (63.8)	0.002
Female	180 (25.2)	135 (22.8)	45 (35.4)	
Race/ethnicity				
Non-Hispanic White	373 (52.1)	325 (55.2)	48 (37.8)	< 0.001
Non-Hispanic Black or African American	52 (7.3)	29 (4.9)	23 (18.1)	
Hispanic, of any race	261 (3.6)	214 (36.3)	47 (37.0)	
Other	30 (4.2)	21 (3.6)	9 (7.1)	
Marital status				
Single	367 (57.3)	320 (58.7)	47 (49.5)	0.050
Relationship	143 (22.3)	119 (21.8)	24 (25.2)	
Married	62 (9.7)	55 (10.1)	7 (7.4)	
Separated, divorced, or widowed	68 (10.6)	51 (9.4)	17 (17.9)	
Sexual orientation				
Heterosexual	579 (85.0)	497 (85.0)	82 (85.4)	0.575
Gay/lesbian	57 (8.4)	51 (8.7)	6 (6.3)	
Bisexual	45 (6.6)	37 (6.3)	8 (8.3)	
Educational attainment				
Some high school or less	93 (14.1)	78 (13.7)	15 (16.5)	0.214
High school/GED	231 (35.1)	192 (33.8)	39 (42.9)	
Some college/technical school	178 (27.0)	160 (28.2)	18 (19.8)	
College graduate	130 (19.7)	116 (20.4)	14 (15.4)	
Advanced degree	27 (4.1)	22 (3.9)	5 (5.5)	
Employment status				
Unemployed > 1 year	222 (33.2)	182 (31.5)	40 (43.5)	
Unemployed < 1 year	178 (26.6)	154 (26.7)	24 (26.1)	0.031
Employed (salary, hourly, or self-employed)	240 (35.9)	218 (37.8)	22 (23.9)	
Disability	29 (4.3)	23 (4.0)	6 (6.5)	
Household income				
$0–$4999	205 (32.4)	156 (29.1)	49 (51.0)	0.001
$5000–$14,999	138 (21.8)	123 (24.6)	15 (15.6)	
$15,000–$29,999	125 (19.8)	109 (20.3)	16 (16.7)	
$30,000–$44,999	75 (11.9)	70 (13.1)	5 (5.2)	
$45,000–$74,999	53 (8.4)	48 (9.0)	5 (5.2)	
> $75,000	36 (5.7)	30 (5.6)	6 (6.3)	
Housing status				
Currently homeless	268 (42.1)	210 (38.7)	58 (61.7)	< 0.001
Not homeless	368 (57.9)	332 (61.3)	36 (38.3)	
How heard about exchange^1^				
Word of mouth	433 (69.5)	354 (67.4)	79 (80.6)	< 0.001
Online	149 (23.9)	141 (26.9)	8 (8.2)	
Fliers/newspaper	30 (4.8)	24 (4.6)	6 (6.1)	
Referral from healthcare provider	11 (17.7)	6 (1.1)	5 (5.1)	
County of residence				
Miami-Dade	497 (72.2)	402 (70.3)	95 (81.9)	0.031
Broward	124 (18.0)	112 (19.6)	12 (10.3)	
Other	67 (9.7)	58 (10.1)	9 (7.8)	

^1^Totals may exceed 100% as participants were allowed to select multiple answers for this question

With regards to injection drug use behavior, participants enrolled at the fixed site and mobile van unit differed significantly by their location of injection drug use, use of alcohol swabs when injecting drugs, reuse of needles and injection equipment, and use of prescription opioids prior to injection drug use. 54.3% of PWID enrolled at the mobile van unit reported most often injecting drugs in a street, park, or parking lot vs 38.2% of PWID enrolled at the fixed site (*p* < .001) (Table 2). In contrast, 51.9% of participants enrolled at the fixed site most often injected in a private home vs 33.7% of participants at the mobile van unit (*p* < .001). Compared to enrollees at mobile site, enrollees at the fixed site reported a greater rate of using alcohol swabs (29.6% vs 14.8%, *p* = .002) and reusing needles/injection equipment (74.8% vs 63.6%, *p* = .003) at least “most of the time” when injecting drugs. 70.5% of PWID enrolled at the mobile van unit reported never using an alcohol swab prior to injecting compared to 44.0% of PWID at the fixed site (*p* = .002). Additionally, 64.3% of participants enrolled at the fixed site reported using prescription opioids before ever initiating injection drug use, compared to 50.0% of mobile van unit participants (*p* = .010). Heroin was the most common drug injected, reported by 82.4% of participants enrolled at the fixed site and 59.8% of participants at the mobile site. Participants enrolled at both sites did not differ significantly by age at first drug injection, types of drugs injected, or needle sharing behavior.

**Table 2:** Drug use behaviors among participants at the Miami IDEA syringe exchange

Characteristics	Sample total *n* (%)	Fixed site *n* (%)	Mobile van unit *n* (%)	*p* value
Total	718 (100.0)	591 (82.3)	127 (17.7)	
Age at first drug injection				
Mean ± standard deviation	24.65 ± 8.80	24.78 ± 8.85	23.70 ± 8.44	0.302
Drugs injected, past month^1^				
Heroin	563 (78.4)	487 (82.4)	76 (59.8)	0.181
Cocaine	187 (26.0)	153 (25.9)	34 (26.8)	
Speedball (heroin and cocaine)	127 (17.7)	110 (18.6)	17 (13.4)	
Methamphetamine	73 (10.2)	68 (11.5)	5 (3.9)	
Fentanyl/carfentanil	71 (9.9)	60 (10.2)	11 (8.7)	
Crack	59 (8.2)	47 (8.0)	12 (9.4)	
Prescription painkillers/opioids	57 (7.9)	52 (8.8)	5 (3.9)	
Other	24 (3.3)	19 (3.2)	5 (3.9)	
Prescription opioid use prior to injection drug use				
Yes	351 (62.0)	306 (64.3)	45 (50.0)	0.010
Location most often inject, past month				
Private home	303 (49.2)	272 (51.9)	31 (33.7)	< 0.001
Street, park, or parking lot	250 (40.6)	200 (38.2)	50 (54.3)	
Public building/restroom	48 (7.8)	43 (8.2)	5 (5.4)	
Other	15 (2.4)	9 (1.7)	6 (6.5)	
Reuse needle/injection equipment when injecting, past month				
None of the time	48 (7.7)	36 (6.8)	12 (13.6)	0.003
Some of the time	118 (19.0)	98 (18.4)	20 (22.7)	
Most of the time	135 (21.8)	109 (20.5)	26 (29.5)	
All of the time	319 (51.5)	289 (54.3)	30 (34.1)	
Shared needle/injection equipment when injecting, past month				
None of the time	411 (63.1)	357 (63.5)	54 (60.7)	0.608
Some of the time	131 (20.1)	111 (19.8)	20 (22.5)	
Most of the time	52 (8.0)	47 (8.4)	5 (5.6)	
All of the time	57 (8.8)	47 (8.4)	10 (11.2)	
Used alcohol swab when injecting, past month				
None of the time	117 (54.9)	55 (44.0)	62 (70.5)	0.002
Some of the time	46 (21.6)	33 (26.4)	13 (14.8)	
Most of the time	21 (9.9)	16 (12.8)	5 (5.7)	
All of the time	29 (13.6)	21 (16.8)	8 (9.1)	

^1^Totals may exceed 100% as participants were allowed to select multiple answers for this question

52.4% of PWID enrolled at the mobile van unit self-reported a prior status of HCV-positive compared to 41.0% of PWID enrolled at the fixed site (*p* = .040) (Table 3). Participants at both sites did not differ significantly by HCV or HIV test result at enrollment, prior HIV status, or HIV treatment status.

**Table 3:** HCV/HIV status among participants at the Miami IDEA syringe exchange

Characteristics	Sample total *n* (%)	Fixed site *n* (%)	Mobile van unit *n* (%)	*p* value
Total	718 (100.0)	591 (82.3)	127 (17.7)	
Prior HCV status				
Negative	340 (54.2)	304 (56.1)	36 (42.9)	0.040
Positive	266 (42.4)	222 (41.0)	44 (52.4)	
Unknown	21 (3.3)	16 (3.0)	5 (5.9)	
HCV test result				
Positive	69 (28.5)	59 (27.6)	10 (35.7)	0.370
Negative	173 (71.5)	155 (72.4)	18 (64.3)	
Prior HIV status				
Negative	584 (88.1)	516 (88.7)	68 (83.95)	0.412
Positive	70 (10.6)	58 (10.0)	12 (14.81)	
Unknown	9 (1.4)	8 (1.4)	1 (1.23)	
HIV test result				
Positive	2 (0.6)	2 (0.70)	0 (0.0)	0.608
Negative	344 (99.4)	304 (99.4)	40 (100.0)	
Currently in HIV treatment, if HIV positive				
Yes	48 (82.8)	39 (83.0)	9 (81.8)	0.927
No	10 (17.2)	8 (17.0)	2 (18.2)	

## Discussion

The main findings of this study suggest that PWID enrolled at the fixed site and mobile van unit varied by sociodemographic and risk characteristics. The mobile van unit seemed to attract PWID from higher risk and harder to reach groups than the fixed site (i.e., more women, more African Americans, higher self-reported baseline HCV seropositivity, lower socioeconomic status, more homelessness, more public injection, less use of alcohol swabs) than the fixed site. There are several possible factors as to why the two modalities attract such varied clientele despite often being in close proximity. One is that the mobile van unit most frequently parks at locations more central to the center of the city’s drug trade about 1 mile from the fixed site location, with the sites separated by the Interstate 95 freeway, a perceived or actual barrier to access. Another is that participants enrolled at the mobile van unit are actively recruited from the surrounding area via in-person engagement with street-level outreach and word-of-mouth to a greater extent than participants enrolled at the fixed site. This result is interesting considering that the mobile van unit enrolled most of its participants in the same neighborhood where the fixed site is located, suggesting that different modalities of syringe exchange programs attract PWID with differing characteristics. These results are consistent with *Riley* et al., (2000) which found that PWID characteristics varied when comparing a pharmacy-based syringe exchange site to a van site in Baltimore, Maryland [17].

These results provide an interesting insight and discussion into the present opioid crisis. Overtown, Miami, is predominantly an African-American neighborhood and has become a hotspot for the purchase of illicit drugs as well as drug overdoses as the opioid crisis has evolved. Interestingly, data collected from this study characterizes the exchange participants as largely white, with the fixed site attracting a larger proportion of white PWID. This is not reflective of the demographics of Overtown nor of PWID in Miami, according to census data and data from the Florida Department of Health, respectively [19, 20]. There has been evidence to suggest that the current opioid crisis, declared a public health emergency, has been notable due to the spread of heroin into predominantly white and middle-class neighborhoods as injection drug use was previously associated with marginalized urban minority populations [21, 22]. Additional data suggesting that fixed site PWID are from a higher socioeconomic status include the fact that nearly 20% of participants at the fixed site come from Broward County, and over one-quarter of PWID at the fixed site found out about the exchange online, evidence that participants at the fixed site may have greater access to transportation and the internet.

Additionally, a significantly greater proportion of PWID enrolled at the fixed site reported using prescription opioids prior to injection drug use. There is evidence to suggest that prior prescription opioid use may contribute to heroin dependence in a subset of PWID, contributing to the current opioid crisis [23, 24]. Our data suggests that the fixed site attracts a greater proportion of those making the progression from prescription opioid use to heroin. Notably, while a significantly greater proportion of PWID enrolled at the fixed site reported using an alcohol swab “most” or “all of the time” when injecting, they also reported a greater rate of reusing needles “all of the time” when injecting, compared to participants enrolled at the mobile van unit. The greater use of alcohol swabs among fixed-site enrolled PWID may reflect greater motivation for safe drug injection behaviors or greater availability to alcohol swabs. However, this safe injection practice does not seem to extend to reuse of needles or injection equipment and likely reflects the effects of drug paraphernalia laws and motivation for public disposal of syringes in Miami due to legal consequences of possession [25]. Overall, for participants enrolled at both sites, there was a high practice of risky injection behaviors, likely due to a preexisting lack of access to clean syringes and lack of harm reduction education prior to the opening of the IDEA Exchange. Such high-risk injection practices among PWID in Miami were shown to lead to significant healthcare expenditures due to bacterial complications of injection drug use prior to the exchange’s opening [26].

Considering the present-day opioid crisis, communities may be looking to decrease their incidence of HIV by increasing access to sterile needles via SEPs. Considering the results of this study combined with the fact that PWID tend to be a hard-to-reach population, implementing mobile-based exchange along with fixed site exchange may increase the scope of an SEP. The current study does not have evidence to support one method over the other, however. Limitations to this study include the differing sample size between those enrolled at the fixed site and mobile van unit as well as the fixed site data including enrollments from December 2016 while the mobile van unit data includes enrollments from its inception in April 2017. Additionally, the data is self-reported and is subject to social desirability bias, although IDEA Exchange staff are well-known and trusted by the PWID community in Miami so there should be a limited effect of this on our results.

This study provides significant conclusions as to the efficacy of mobile van units in targeting hard to reach populations in urban areas. Importantly, it provides further evidence on how best to reach those affected by the national opioid crisis, especially in states that have been late to adopt syringe exchange. Further studies will explore whether convenience or other factors such as transportation or stigma affect the sociodemographic makeup of the exchange participants. As the evidence regarding the efficacy of syringe exchange literature is scientific fact, and more states pass syringe exchange laws to combat this crisis, varied efforts to target PWID should be made. The present study suggests that multimodal delivery of syringe services to PWID may be vital to reducing the spread of HIV and other communicable diseases in light of our continuing opioid crisis.
